# The Sydney Triage to Admission Risk Tool (START) to predict Emergency Department Disposition: A derivation and internal validation study using retrospective state-wide data from New South Wales, Australia

**DOI:** 10.1186/s12873-016-0111-4

**Published:** 2016-12-03

**Authors:** Michael M. Dinh, Saartje Berendsen Russell, Kendall J. Bein, Kris Rogers, David Muscatello, Richard Paoloni, Jon Hayman, Dane R. Chalkley, Rebecca Ivers

**Affiliations:** 1 Emergency Department, Royal Prince Alfred Hospital, Sydney, NSW Australia; 2 Discipline of Emergency Medicine, The University of Sydney, Sydney, NSW Australia; 3 Faculty of Nursing, The University of Sydney, Sydney, NSW Australia; 4 The George Institute for Global Health, The University of Sydney, Sydney, NSW Australia; 5 School of Public Health and Community Medicine, University of New South Wales, Sydney, NSW Australia; 6 Health Education and Training Institute, New South Wales Ministry of Health, Sydney, NSW Australia; 7 School of Nursing and Midwifery, Flinders University, Adelaide, South Australia Australia; 8 Emergency Department, Royal Prince Alfred Hospital, Missenden Rd, Camperdown, NSW 2050 Australia

**Keywords:** Decision, Risk score, Triage

## Abstract

**Background:**

Disposition decisions are critical to the functioning of Emergency Departments. The objectives of the present study were to derive and internally validate a prediction model for inpatient admission from the Emergency Department to assist with triage, patient flow and clinical decision making.

**Methods:**

This was a retrospective analysis of State-wide Emergency Department data in New South Wales, Australia. Adult patients (age ≥ 16 years) were included if they presented to a Level five or six (tertiary level) Emergency Department in New South Wales, Australia between 2013 and 2014. The outcome of interest was in-patient admission from the Emergency Department. This included all admissions to short stay and medical assessment units and being transferred out to another hospital. Analyses were performed using logistic regression. Discrimination was assessed using area under curve and derived risk scores were plotted to assess calibration.

**Results:**

1,721,294 presentations from twenty three Level five or six hospitals were analysed. Of these 49.38% were male and the mean (sd) age was 49.85 years (22.13). Level 6 hospitals accounted for 47.70% of cases and 40.74% of cases were classified as an in-patient admission based on their mode of separation. The final multivariable model including age, arrival by ambulance, triage category, previous admission and presenting problem had an AUC of 0.82 (95% CI 0.81, 0.82).

**Conclusion:**

By deriving and internally validating a risk score model to predict the need for in-patient admission based on basic demographic and triage characteristics, patient flow in ED, clinical decision making and overall quality of care may be improved. Further studies are now required to establish clinical effectiveness of this risk score model.

## Background

One of the most important aspects of treating patients in Emergency Departments (ED) is deciding whether a patient is safe for discharge or requires in-patient admission for further treatment and stabilisation [1]. These are termed disposition decisions and they involve the complex interaction of clinical factors such as diagnoses, severity and response to treatment, as well as social and clinician factors. It has been shown that most experienced ED clinicians use clinical judgement to make disposition decisions, however in ED, these are often made in the context of various time pressures, limited clinical information and ED overcrowding which can lead to clinical errors [1, 2].

Clinical decisions can also take time, particularly when there are multiple patients with varying acuity, requiring multiple and simultaneous decisions. A study of process factors in ED demonstrated that discharge and in-patient admission disposition decisions take on average around 130 min and 200 min from ED arrival time respectively [2]. The need for timely accurate and safe decision making in ED has grown more urgent with the increased demand for Emergency Department services, the ageing population, limited in-patient bed capacity and time based ED performance targets [3–5]. Overcrowding in EDs has become a major public health concern in Australia and around the world. One innovative solution involves the use of data analytics and decision support systems to assist clinicians at the point of triage and bedside. A recent study of four hospitals, including two Veteran’s Affairs hospitals in the US developed a prediction model for admission using urgency categories, presenting problem categories and age, with moderately high accuracy (Area Under Receiver Operator Characteristic Curve between 0.80 to 0.89) [6].

We sought to derive and internally validate a similar model using State-wide Emergency data in Australia, which will ultimately be used to develop and implement a risk score base decision support tool for disposition at point of triage. The objective was to determine if similar measures of overall model accuracy could be obtained using a more generalised dataset containing many ED’s. The driver of the project is to translate data based research into decision support tools that not only assist with process efficiency but also improve the quality of clinical decision making and potentially facilitate shared decision making between clinicians and patients. This may be translated into tools that improve streaming to medical assessment or short stay units, assist clinicians in prioritising the clinical workup for ED patients that have a high probability of being admitted or discharged.

## Methods

### Design

This was a retrospective data analysis of State-wide Emergency Department data and undertaken as part of the Demand for Emergency Services Trend in Years 2010–15 (DESTINY) study [7].

### Setting

New South Wales is the most populous state in Australia with a population of around 7.5 million people and a land area of 809, 000 km^2^ [8].

### Data sources

The Emergency Department Data Collection (EDDC) Registry routinely collects patient level data on presentations to all designated Emergency Departments in New South Wales (NSW). Data collection includes, referral source (self-referred, General Practice, Specialist, Nursing Home), mode of arrival (self-referral, Ambulance), hospital facility, triage category (Australasian Triage Scale) [9], presenting problem, mode of separation (admitted to hospital, discharged or died). Presenting problems allocated by triage nurses at the point of patient arrival to ED were categorised into broad clinical groups and described elsewhere (see Table 1) [7]. For example, neurological complaints included headache, dizziness, weakness and ataxia), respiratory complaints included shortness of breath, cough and wheeze and cardiovascular complaints included chest pain and palpitations. The time that a patient was triaged was used as the ED arrival time and categorised using commonly accepted ED clinician shift times (0800–1759, 1800–2359 and 0000–0759).

**Table 1:** Baseline characteristics of admitted and non-admitted patients in the derivation dataset (*N* = 860832)

Variable	Non admitted *N* = 510435	Admitted*N* = 350397	*P* value
Age (%)			<0.001
16–19 yrs	43187 (8.46)	11555 (3.30)	
20–39 yrs	217827 (42.67)	72519 (20.7)	
40–59 yrs	137922 (27.02)	82491 (23.54)	
60–79 yrs	82207 (16.11)	105495 (30.11)	
≥80 yrs	29292 (5.74)	78337 (22.36)	
Male (%)	251571 (49.29)	173218 (49.43)	0.17
Indigenous (%)	21766 (4.26)	9767 (2.79)	<0.001
Nursing home (%)	3229 (0.63)	9926 (2.83)	<0.001
Ambulance arrival (%)	105123 (20.59)	181714 (51.86)	<0.001
Triage category (%)			<0.001
1	726 (0.14)	8606 (2.46)	
2	43046 (8.43)	95212 (27.17)	
3	156133 (30.59)	162974 (46.51)	
4	242420 (47.49)	78319 (22.35)	
5	68101 (13.34)	5285 (1.51)	
Previous ED presentation within 7 days (%)	47144 (9.24)	32956 (9.41)	0.008
Admission within 30 days	22220 (4.35)	40997 (11.70)	<0.001
Hour of presentation (%)			<0.001
0800–1759	257400 (50.43)	189603 (54.11)	
1800–2259	174097 (34.11)	106963 (30.53)	
2300–0759	78938 (15.46)	53831 (15.36)	
Presenting problem type (%)			<0.001
Abdominal, gastrointestinal	63630 (12.47)	60845 (17.36)	
Cardiovascular	45068 (8.83)	54968 (15.69)	
General symptoms	43702 (8.56)	37027 (10.57)	
Febrile illness	11460 (2.25)	14522 (4.14)	
Injury	106890 (20.94)	42736 (12.20)	
Respiratory	19370 (3.79)	32264 (9.21)	
Musculoskeletal	57727 (11.31)	20157 (5.75)	
Neurological	30750 (6.02)	32569 (9.29)	
Mental health	24020 (4.71)	15859 (4.53)	
Toxicological	4893 (0.96)	4150 (1.18)	
ENT/eye/head and neck	35014 (6.86)	5277 (1.51)	
Administrative	18589 (3.64)	4282 (1.22)	
Genitourinary	16318 (3.20)	11864 (3.39)	
Social	302 (0.06)	310 (0.09)	
Endocrine	1133 (0.22)	1903 (0.54)	
Obstetrics, Gynaecology	14124 (2.77)	3675 (1.05)	
Skin, allergy	17253 (3.38)	7041 (2.01)	
Other medical	192 (0.04)	948 (0.27)	

Hospital facilities were classified according to current Ministry of Health definitions for designation of Emergency Departments based on case-mix, staffing and specialist facilities within each hospital [10]. In brief, these range from Level six centres comprising tertiary level teaching hospital that are Major Trauma Centres (including two specialist paediatric centres), Level five centres tertiary level non trauma centres, Level four centres which are mainly Metropolitan District level hospitals, Level three centres which are smaller district and general hospitals, and level two and one centres which comprise smaller rural multi-purpose and urgent care centres. Estimated Residential Population by age and sex were obtained from the Australian Bureau of Statistics [8].

### Inclusion criteria

Adult patients (age ≥ 16 years) were included if they presented to a Level 5 or 6 Emergency Department between 2013 and 2014. Patients who were dead on arrival or were planned representations were excluded as were patients who did not wait for triage and had presenting problem field entries that were either missing or uninterpretable.

### Primary outcome

The outcome of interest was in-patient admission from the Emergency Department. This included all admissions to short stay and medical assessment units and being transferred out to another hospital.

### Statistical analyses

Descriptive statistics were used to compare univariate predictors of in-patient admission. The dataset was then randomly split into derivation and validation datasets. Logistic regression with stepwise selection was used to determine predictors in the derivation dataset which was then tested on the validation dataset to determine Area Under Curve (AUC) of Receiver Operator Characteristic (ROC) curves. Reference values for variables were assigned through investigator consensus. Risk scores were assigned based on variable coefficient values and predicted versus observed risk of admission for each score were plotted to obtain calibration curves. The procedure was then repeated using least absolute shrinkage and selection operator (LASSO) regression as an alternative to variable selection.

### Ethics

Access to data was approved by the NSW Population Health Services Research Ethics Review Committee.

## Results

### Study population

A total of 1,773,550 ED presentations were identified of which 52,256 cases (2.95%) were excluded. Of the excluded cases, 26,915 had missing presenting problems, 24,564 (1.41%) had presenting problems that were uncodeable and 777 cases did not wait for triage, leaving 1,721,294 presentations from twenty three level 5 or 6 hospitals. Of these 49.38% were male and the mean (sd) age was 49.85 years (22.13). Level 6 hospitals accounted for 47.70% of cases and 40.74% of cases were classified as an in-patient admission based on their mode of separation.

### Univariable and multivariable predictors of in-patient admission

The dataset was randomly allocated so that 860,832 cases (50.01%) were assigned to the derivation dataset. Table one compares the baseline characteristics of admitted and non-admitted ED patients in the derivation dataset. Patients requiring admission were older with higher triage acuity scores, and associated with cardiovascular, respiratory febrile illness and other general medical presenting problems and had a previous in-patient admission within 30 days of current ED presentation.

### Model performance

After excluding non-significant variables in univariate analysis, the final multivariable model including age, arrival by ambulance triage category previous admission and presenting problem (Table 2) had a AUC of 0.82 (95% CI 0.81, 0.82) and with a Hosmer-Lemeshow test statistic *p* < 0.001 for calibration. When this was repeated using LASSO selection, the AUC was 0.81 (95% CI 0.814, 0.816).

**Table 2:** Multivariable model of in-patient admission with risk score using derivation set Akiake Information Criterion (intercept only 2326760, intercept and covariates 1768771) Area under Receiver Operator curve for validation dataset 0.82 (95% CI 0.81, 0.82). Hosmer-Lemeshow test statistic *p* < 0.001

Variable	Coefficient	Odds ratio (95% CI)	*P* value	Risk score
Age				
16–19 yrs	Ref	Na		0
20–39 yrs	0.19	1.21 (1.19,1.23)	<0.001	+1
40–59 yrs	0.61	1.85 (1.82, 1.88)	<0.001	+3
60–79 yrs	1.20	3.31 (3.25, 3.37)	<0.001	+6
≥80 yrs	1.79	6.01 (5.89, 6.13)	<0.001	+9
Ambulance arrival	0.77	2.17 (2.15, 2.19)	<0.001	+4
Triage category				
1	4.47	87.13 (82.15, 92.60)	<0.001	+24
2	2.99	19.84 (19.28, 20.32)	<0.001	+16
3	2.08	7.97 (7.80, 8.15)	<0.001	+11
4	1.10	3.00 (2.94, 3.07)	<0.001	+5
5	Ref	Na		0
Admission within 30 days	0.66	1.93 (1.90, 1.96)	<0.001	+3
ED arrival time				
0800–1759	0.21	1.23 (1.22, 1.25)	<0.001	+1
1800–2259	0.01	1.01 (1.00, 1.02)	0.06	0
2300–0759	Ref	na		0
Presenting problem				
Abdominal, gastrointestinal	0.33	1.39 (1.37, 1.41)	<0.001	+2
Cardiovascular	−0.71	0.49 (0.46, 0.48)	<0.001	−3
General symptoms	Ref	Na		0
Febrile illness	0.65	1.91 (1.87, 1.96)	<0.001	+3
Injury	−0.75	0.47 (0.46, 0.49)	<0.001	−4
Respiratory	0.01	1.01 (0.99,1.03)	0.19	0
Musculoskeletal	−0.57	0.56 (0.55, 0.57)	<0.001	−3
Neurological	−0.25	0.78 (0.77, 0.79)	<0.001	−1
Mental health	−0.32	0.72 (0.71, 0.74)	<0.001	−2
Toxicological	−0.30	0.74 (0.72, 0.77)	<0.001	−2
ENT/eye/head and neck	−1.17	0.31 (0.30, 0.32)	<0.001	−6
Administrative	−0.57	0.57 (0.55, 0.59)	<0.001	−3
Genitourinary	−0.16	0.85 (0.83, 0.87)	<0.001	−1
Social	0.19	1.21 (1.07, 1.38)	0.004	+1
Endocrine	−0.03	0.97 (0.91, 1.05)	0.26	0
Obstetrics, Gynaecology	−0.55	0.58 (0.56, 0.59)	<0.001	−3
Skin, allergy	−0.30	0.74 (0.72, 0.76)	<0.001	−2
Other medical	0.99	2.70 (2.40, 3.04)	<0.001	+5

**Fig. 1:**
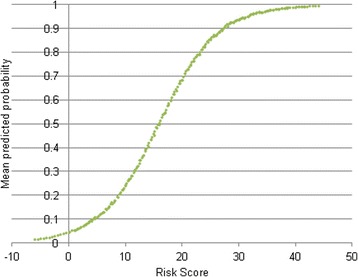
Mean predicted probability of in-patient admission based on all possible risk score totals

**Fig. 2:**
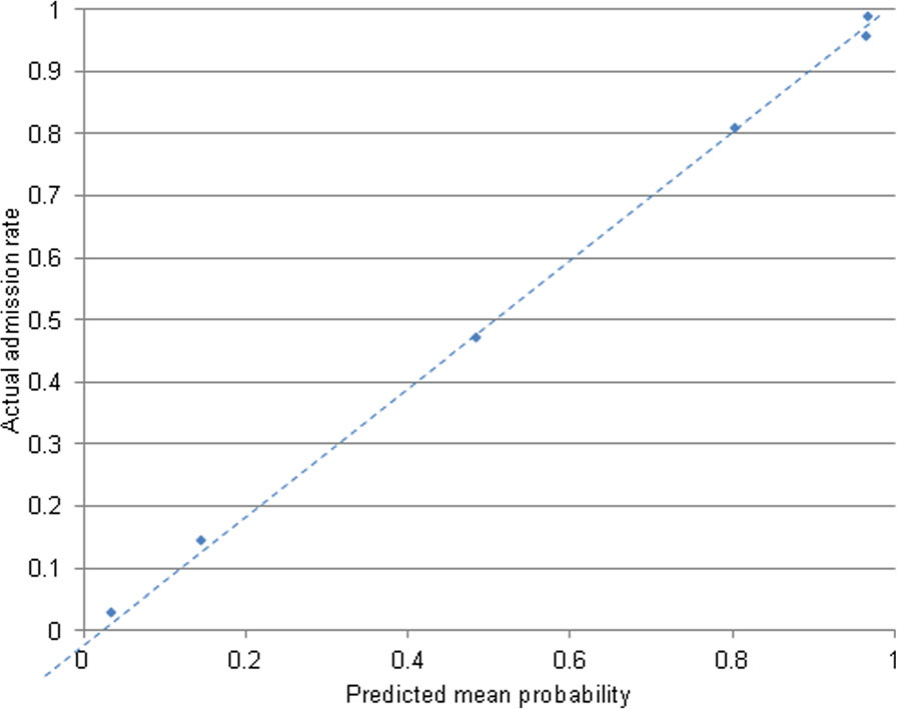
Calibration curve of actual admission rate by predicted mean probability - dots denoting each risk score category (total risk score >40, 30–40, 20–30, 10–20, 1–10, <1). Dotted line denotes perfect calibration

Figure 1 shows the range of risk scores possible with mean admission rates. Deciles of risk score ranges with corresponding mean predicted probabilities of in-patient admission were as follows: Risk score <1 (3%), risk score 1–10 (14%), risk score 11–20 (47%), 20–30 (81%), 30–40 (96%), >40 (99%). These were plotted on a calibration curve is shown in Fig. 2. Over high risk score ranges (risk score >20) the positive predictive value was 86.8%, negative predictive value of 64.25% for in-patient admission. For low risk scores (risk score <10), the positive predictive value for discharge from ED was 89.85% and the negative predictive value was 46.28%. The optimum point on the ROC curve corresponded to a sensitivity of 88% and a specificity of 67% corresponding to a risk score of 13.

## Discussion

This study demonstrates that disposition prediction in ED can be made with reasonable accuracy using only initial presenting problem together with known variables such as age, mode and time of arrival. The risk scores can be summed to obtain an estimate of the risk of inpatient admission or discharge from the emergency department. The authors have named this model the “Sydney Triage to Admission Risk Tool” (START) and is the first such study reported in Australia. The overall accuracy of the model was around 82% with an AUC comparable to previously published clinical risk scores such as the Pneumonia Severity Index and previous data analysis studies of ED disposition from US hospitals [6, 11]. The Hosmer-Lemeshow test statistic suggested that calibration was suboptimal, however this may be a function of the large sample size – indeed the calibration curve showed that any lack of calibration was unlikely to be clinically meaningful.

This study has a number of advantages in that it used state-wide data for all tertiary hospitals across NSW making it applicable across many centres, utilises a small number of variables that can be reliably obtained upon patient arrival by clerical and triage staff in ED. Used in this way, the derived risk score may potentially be used to rapidly identify patients suitable either for immediate in-patient bed allocation (high risk), further assessment in a medical or surgical assessment area (moderate risk), or streamed to a fast track area for those with anticipated discharge (low to negligible risk). In doing so, the time to disposition for patients in ED can be reduced resulting in improved patient flow and efficiency. It may also be used as a decision support at the bedside, perhaps integrated within clinical information systems or mobile devices to assist clinicians managing patients to safely decide where the most suitable destination for the patient should be [12].

Further studies are now warranted to evaluate this data modelling study and see if model performance translates in actual clinical practice and reduces ED decision times. These are currently being planned in New South Wales and will evaluate the risk score based on the performance reported in this study. Studies are also required to compare model performance against clinician based prediction. One single centre study from Westmead Hospital demonstrated that senior clinicians predicted ED discharge with a positive predictive value of around 0.90 however the positive predictive value for in-patient admission prediction was only around 55% [13]. A study from Queensland used data analytics to predict ED demand based on day of week and previous total ED presentations with the aim of forecasting bed requirements on an administrative level [14]. In contrast this study examined individual factors that predicted disposition with the aim of improving patient flow in ED. Increasing efficiency in ED has been shown to improve quality of care in ED and in-hospital mortality [15, 16]. ED performance is constrained by growing demand, overcrowding and a relatively junior and rotating workforce particularly after hours [15, 16]. These conditions are ideal for risk score based decision tools designed to reduce human factor variation in clinical decision making and to stream patients reliably into different clinical areas.

The study also provides a useful reference point for more advanced data analytic methods such as neural networks to investigate whether model performance can be improved by including facility and specific subgroups of presenting problems. There are around 400 different presenting problems in the dataset, and the major problem with categorising presenting problems as we have done is the overlap between different presenting problems. Shortness of breath for instance can be both a cardiovascular and respiratory complaint. Using more sophisticated machine learning techniques opens the possibility of more refined prediction based on "big data" principles of using specific personal and presentation characteristics, including the use of historical background information, previous diagnoses and presenting problems linked to previous admissions which cannot be analysed using more traditional statistical methods as we have done [17]. Advantages of this approach include the inclusion and analysis of more granular decision making nodes such as the most appropriate clinical service and the most appropriate clinical service location to admit to such as the Intensive Care unit or normal in-patient ward location. It may also also account for the observation that most presenting problem types actually decreased the odds of in-patient admission and this was perhaps due to the combining of fairly disparate presenting problems under broad categories, for instance “cough” and “cyanosis” were both classified under respiratory problems, even though they represent two extremes of respiratory conditions.

It should be noted that the in-patient admission rate of around 40% includes those patients admitted to Emergency Department short stay and observation units, as they are classified under current administrative definitions as an in-patient unit. Notwithstanding this, the in-patient admission rate is still substantially higher than previously reported [3] in other regions in Australia and requires further investigation. The most likely explanation being that this study included higher level tertiary centres that are more likely to treat more complex patients. Similarly it is unclear why ED arrival time during normal business hours should be more predictive of in-patient admission compared to other hours of the day. It may reflect bed management and hospital specific practices with more referrals from specialty and general practice clinics during the day or it may be a reflection of the types of patients presenting after hours. The impact of in-patient ward availability (access block) on disposition outcomes was also not evaluated in this study. Hospital overcrowding may have a confounding effect on disposition decisions by clinicians, reducing the likelihood of admission in a given day if it is already known that there are no beds in the hospital available. This requires prospective evaluation, which is currently underway.

Other acknowledged limitations include the lack of information of background medical history, which can often be crucial in making disposition decisions. This can potentially be accessed by linkage with admitted patient databases and the use of a cumulative list of previous ED diagnoses within this dataset. It is also difficult to quantify the role of clinical experience and clinical judgement in making disposition decisions and the overall misclassification rate of 18% underscores the importance of those clinical factors, therefore as with any clinical decision rule this model should not be used in isolation but in conjunction with clinical acumen.

Although we have only used data from tertiary hospitals, the majority of hospitals EDs in NSW are smaller non tertiary hospitals. Including such centres may have introduced heterogeneity due to varying triage and admission practices as well as differences in patient presentation patterns. Therefore separate or multilevel analyses are required to incorporate all types of ED’s.

## Conclusion

In conclusion, we have derived and internally validated a risk score model to predict the need for in-patient admission based on basic demographic and triage characteristics. This model may be used to facilitate patient flow in ED, standardise clinical decision making and improve overall quality of care. Further translational studies are now warranted to assess model performance in clinical practice and evaluate its impact on patient outcomes.

## Abbreviations

ED: Emergency Department
US: United States of America
AUC: Area Under Curve
ROC: Receiver Operator Characteristic
EDDC: Emergency Department Data Collection Registry
